# Endoscopic gallbladder inside-stenting combined with aspirated lavage for calculous cholecystitis in poor surgical candidates: a prospective pilot study

**DOI:** 10.1038/s41598-023-48543-1

**Published:** 2023-11-30

**Authors:** Tadahisa Inoue, Rena Kitano, Mayu Ibusuki, Yuji Kobayashi, Kiyoaki Ito, Masashi Yoneda

**Affiliations:** Department of Gastroenterology, Aichi Medical University, 1-1 Yazakokarimata, Nagakute, Aichi 480-1195 Japan

**Keywords:** Cholecystitis, Cholelithiasis

## Abstract

Although long-term stent placement via endoscopic gallbladder stenting (EGBS) reportedly reduces cholecystitis recurrence in patients unfit to undergo cholecystectomy, it can increase the frequency of other late adverse events (AEs) such as cholangitis. This study aimed to examine the feasibility of endoscopic gallbladder inside-stenting (EGB-IS) with lavage and aspiration. This prospective, single-center, pilot study enrolled 83 patients with acute calculous cholecystitis who were poor candidates for surgery. A dedicated catheter with eight side holes was used for lavage and aspiration, and a dedicated single-pigtail stent equipped with a thread was used for EGB-IS. Outcomes such as technical success, clinical success, early AEs, recurrence of cholecystitis, and other symptomatic late AEs associated with EGB-IS with lavage and aspiration were evaluated. The technical and clinical success rates were 80.7% (67/83) and 98.5% (66/67), respectively. The rate of early AEs was 3.6% (3/83). The rate of recurrent cholecystitis was 4.5% (3/66) and that of symptomatic late AEs (besides cholecystitis) was 6.1% (4/66). Consequently, the rate of overall late AEs (cholecystitis plus other events) was 10.6% (7/66). The 1-, 2-, and 3-year cumulative incidence rates of all late AEs were 3.2%, 11.2%, and 18.9%, respectively. EGB-IS with lavage and aspiration for calculous cholecystitis showed promising results in poor surgical candidates. EGB-IS may be useful when EGBS with long-term stent placement is planned, since prevention of cholecystitis recurrence, without a rise in the incidence of other AEs, is anticipated.

## Introduction

Calculous cholecystitis is a common disease, for which early surgical cholecystectomy is the standard treatment^1^. However, either percutaneous or endoscopic gallbladder drainage is indicated when emergent surgery cannot be performed in the acute phase due to the patient’s general condition^2^. Even in such cases, the recurrence rate is high if cholecystectomy is not performed after drainage; thus, elective surgery is recommended after improvement in infection, inflammation, and the patient’s general condition^3–8^. However, owing to advanced age and/or significant underlying diseases, cholecystectomy may be difficult in some cases, even in the elective setting^9,10^.

Endoscopic gallbladder stenting (EGBS) via endoscopic retrograde cholangioscopy does not require an external fistula tube, unlike percutaneous transhepatic gallbladder drainage (PTGBD); therefore, long-term stent placement is possible without affecting the patient’s daily life. Several studies have demonstrated that long-term stent placement via EGBS is useful for preventing the recurrence of cholecystitis^11–13^. However, studies have also shown that the frequency of other late adverse events (AEs), such as cholangitis, is elevated due to duodenobiliary reflex and bacterial contamination via the stents, especially when the period of stent placement is prolonged^14^. Another concern with EGBS is that the highly viscous, infected bile sometimes causes premature obstruction and insufficient drainage, unlike PTGBD.

To overcome these problems, we developed a novel method of endoscopic gallbladder inside-stenting (EGB-IS), in which the stent is placed above the duodenal papilla, combined with lavage and aspiration, to achieve better short- and long-term outcomes after EGBS. This study aimed to examine the feasibility and utility of EGB-IS with lavage and aspiration for calculous cholecystitis in poor surgical candidates.

## Methods

### Study design and population

This prospective, single-center, pilot study included consecutive patients with acute calculous cholecystitis who were considered poor surgical candidates and scheduled to undergo EGB-IS with lavage and aspiration. The study period extended from July 2017 to December 2022. Poor surgical candidates were defined as patients who satisfied at least one of following criteria based on previous research^15^: aged ≥ 80 years, with an American Society of Anesthesiology Physical Status (ASA-PS) grade 3 or higher, Karnofsky Performance Status < 50, and/or age-adjusted Charlson Comorbidity Index (CCI) scores ≥ 5^16^; however, the feasibility of surgical cholecystectomy (including elective cholecystectomy after improvement) was determined on the basis of discussion with surgeons at diagnosis. The exclusion criteria were age < 20 years, difficulty in conducting endoscopic procedures, presence of biliary stricture, concomitant biliary malignancy, and refusal to participate in the study.

The Institutional Review Board of Aichi Medical University Hospital approved this study, which was conducted in accordance with the principles of the Declaration of Helsinki (Approval Number: 2017-H129). All patients provided written informed consent before the procedure. The study protocol was registered with the University Hospital Medical Information Network Clinical Trial Registry database (identifier: UMIN000028074).

### Devices

**Figure 1:**
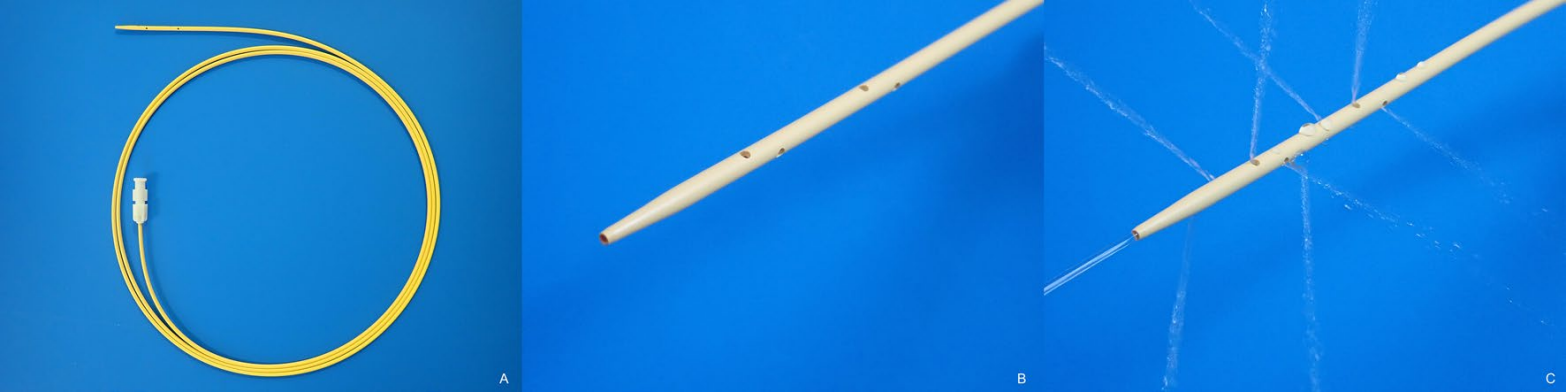
A dedicated 7-Fr catheter for lavage and aspiration. (**A**) The tip of the catheter was highly tapered to reduce the gap with the guidewire, and eight side holes were created within 25 mm of the tip. (**B**) This structure achieves excellent bile aspiration and high-flow washing with saline. (**C**) photograph of spraying saline.

A dedicated 7-Fr catheter (Gadelius Medical, Tokyo, Japan), which fits a 0.025- or 0.035-inch guidewire, was used for lavage and aspiration (Fig. 1). The tip of the catheter is highly tapered to reduce the gap between the catheter and guidewire. Eight side holes with a diameter of 1 mm were created within a length of 25 mm from the tip, allowing aspiration and lavage with high flow.

**Figure 2:**
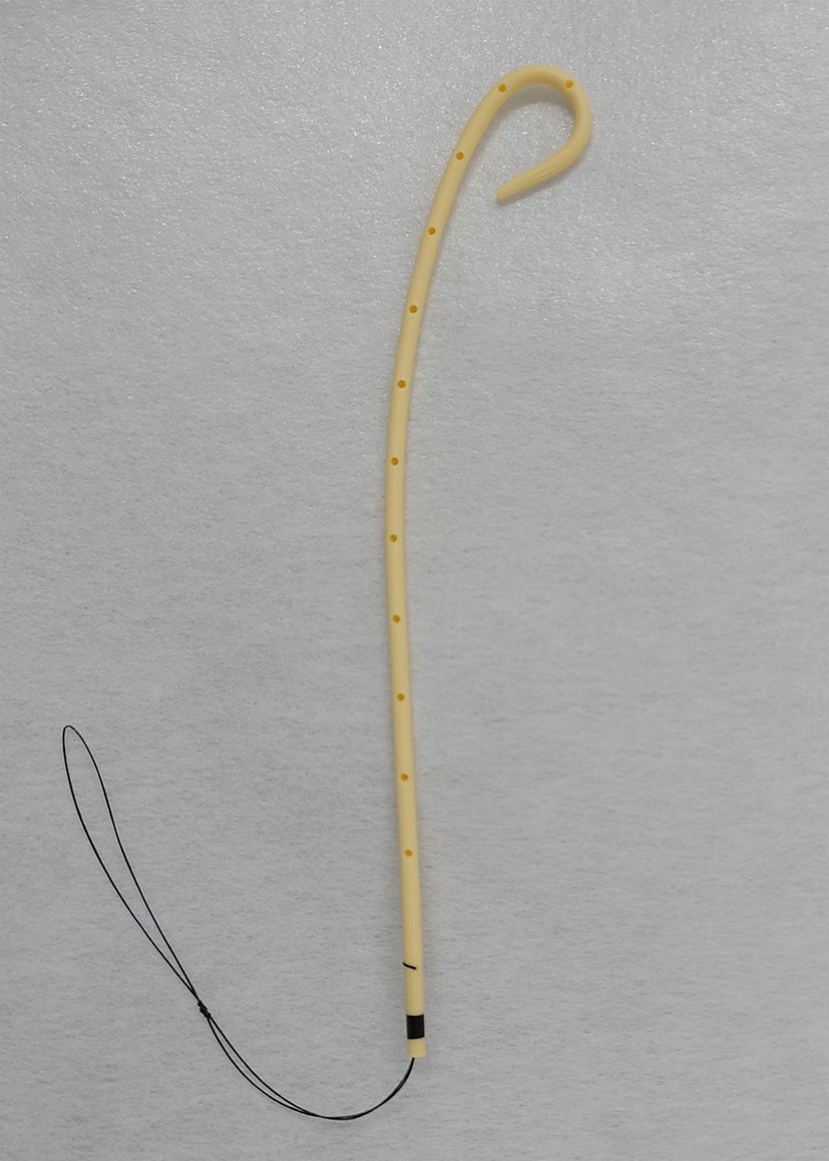
A dedicated 7-Fr, single pigtail, plastic stent for endoscopic gallbladder inside-stenting. It is shaped like a single pigtail on the gallbladder side and the tip is highly tapered for good insertability. The bile duct side is straight and a retrieval thread is attached to the distal stent end.

A dedicated 7-Fr, single-pigtail, plastic stent (Hanako Medical, Saitama, Japan) was used for EGB-IS, which resembles a single pigtail on the gallbladder side and is straight on the bile duct side (Fig. 2). This stent can be used with both 0.025- and 0.035-inch guidewires, and the tip is highly tapered for good insertability. The stent length, which is the distance from the base of the pigtail part to the distal end, can be set to 90, 110, or 130 mm. A 90-mm long Nylon retrieval thread was attached to the distal end of the stent, so that the stent could be retrieved easily by pulling this thread.

### Technique

**Figure 3:**
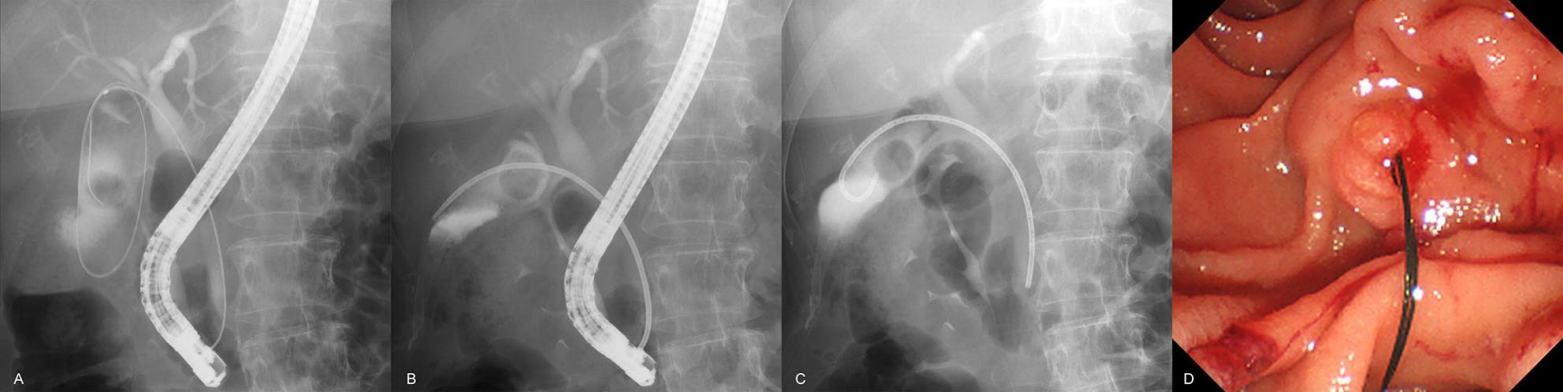
After successful placement of the guidewire in the gallbladder (**A**), a dedicated catheter was inserted over the guidewire. Subsequently, the guidewire was removed temporarily, and aspiration of as much bile as possible and meticulous lavage with approximately 100 mL saline were conducted (**B**). Finally, the guidewire was reinserted, followed by removal of the catheter, and deployment of the dedicated 7-Fr single-pigtail stent from the gallbladder to the bile duct (**C**). The thread was hung into the duodenum through the duodenal papilla (**D**).

A standard duodenoscope (Olympus Medical Systems, Tokyo, Japan) was inserted into the duodenum, followed by biliary cannulation. Choledocholithiasis, if present, was eliminated using a retrieval balloon and basket after endoscopic sphincterotomy. Otherwise, endoscopic sphincterotomy was not performed. Subsequently, guidewire insertion into the gallbladder was attempted via the cystic duct using a standard cannula and an angle-tip guidewire. If the access to the gallbladder was difficult, another angled-tip guidewire, swing-tip cannula-guided technique, and/or cholangioscopy-guided technique^17,18^ was employed. After the successful placement of the guidewire in the gallbladder, a dedicated catheter was inserted over the guidewire. After the guidewire was removed temporarily, bile was aspirated as much as possible, and lavage was performed meticulously with approximately 100 mL of saline. Finally, the guidewire was reinserted, followed by removal of the catheter. Subsequently, a dedicated 7-Fr, single-pigtail, stent was placed from the gallbladder to the bile duct. The thread was hung into the duodenum through the duodenal papilla (Fig. 3). The procedure was performed urgently (within 24 h). Elective stent exchange and removal were not performed.

When an AE occurred and the stent had to be removed, the distal end of the stent was pulled out into the duodenum by grasping and tugging at the thread using forceps. The stent was subsequently grasped using forceps and pulled and removed through the scope channel.

An endoscopist with experience in endoscopic retrograde cholangiography and EGBS conducted or directly supervised all procedures.

### Follow-up

Blood tests were performed on the day after the procedure; computed tomography was performed, as appropriate, when AE was suspected. After achieving clinical success, patients underwent regular follow-up with or without abdominal radiography at least annually or semiannually. During this time, they were instructed to visit the hospital immediately if they experienced symptoms, including abdominal pain. Blood tests and imaging studies, such as ultrasonography and computed tomography, were performed upon suspicion of late AEs including recurrent cholecystitis.

### Outcomes and definitions

The outcomes included technical success, clinical success, early AEs, cholecystitis recurrence, and other symptomatic late AEs associated with EGB-IS with lavage and aspiration. Technical success was defined as successful completion of biliary cannulation, advancement of the guidewire into the gallbladder, insertion of the dedicated catheter, appropriate lavage and aspiration, and stent placement above the papilla. Clinical success was defined as the resolution of clinical symptoms and laboratory findings associated with cholecystitis without the need for additional drainage procedures. The diagnosis and severity of cholecystitis and cholangitis were based on the Tokyo Guidelines 2018^19,20^. Other AEs were defined and graded according to the criteria provided by the American Society for Gastrointestinal Endoscopy^21^.

### Statistical analysis

The sample size was not calculated because this was a pilot investigation of a novel procedure using novel devices. Categorical variables were presented as numbers and percentages, and continuous variables were expressed as medians and interquartile ranges (IQR). The Kaplan–Meier method was used to estimate the cumulative incidence of the AEs. Patients’ data were censored if they did not experience any AE until the time of death, transfer to another institution, or the end of the study period. Additionally, if elective surgical cholecystectomy was performed after improvement of the general condition, the patient’s data were also censored at that time. All statistical analyses were conducted using EZR version 1.61 (Saitama Medical Center, Jichi Medical University, Saitama, Japan)^22^.

## Results

### Patient characteristics

Eighty-three patients who met the eligibility criteria were enrolled in this study. Table 1 presents the patients’ characteristics, including sex, age, age-adjusted CCI, ASA-PS classification, cause and severity of cholecystitis, number and diameter of the gallstones, and presence of choledocholithiasis. The median follow-up period was 603 (IQR 404–1314) days.

**Table 1:** Baseline characteristics of the participants.

Number of patients, n	83
Sex (male/female), n	49/34
Median age, (IQR) (years)	81 (75–86)
Age-adjusted CCI score, n (%)	
≥ 5	62 (74.7)
ASA-PS classification, n (%)	
≥ 3	56 (67.5)
Cause of cholecystitis, n (%)	
Calculus	83 (100)
Severity of cholecystitis, n (%)	
Mild	22 (26.5)
Moderate	50 (60.2)
Severe	11 (13.3)
Number of cholelithiasis, n (%)	
≥ 3	32 (38.6)
Median diameter of the maximum cholelithiasis, (IQR) (mm)	10 (7–14)
Presence of choledocholithiasis, n (%)	31 (37.3)
Median follow-up duration, (IQR) (days)	603 (404–1314)

*IQR* interquartile range, *CCI* Charlson comorbidity index, *ASA-PS* American Society of Anesthesiology Physical Status.

### Procedural outcomes

**Figure 4:**
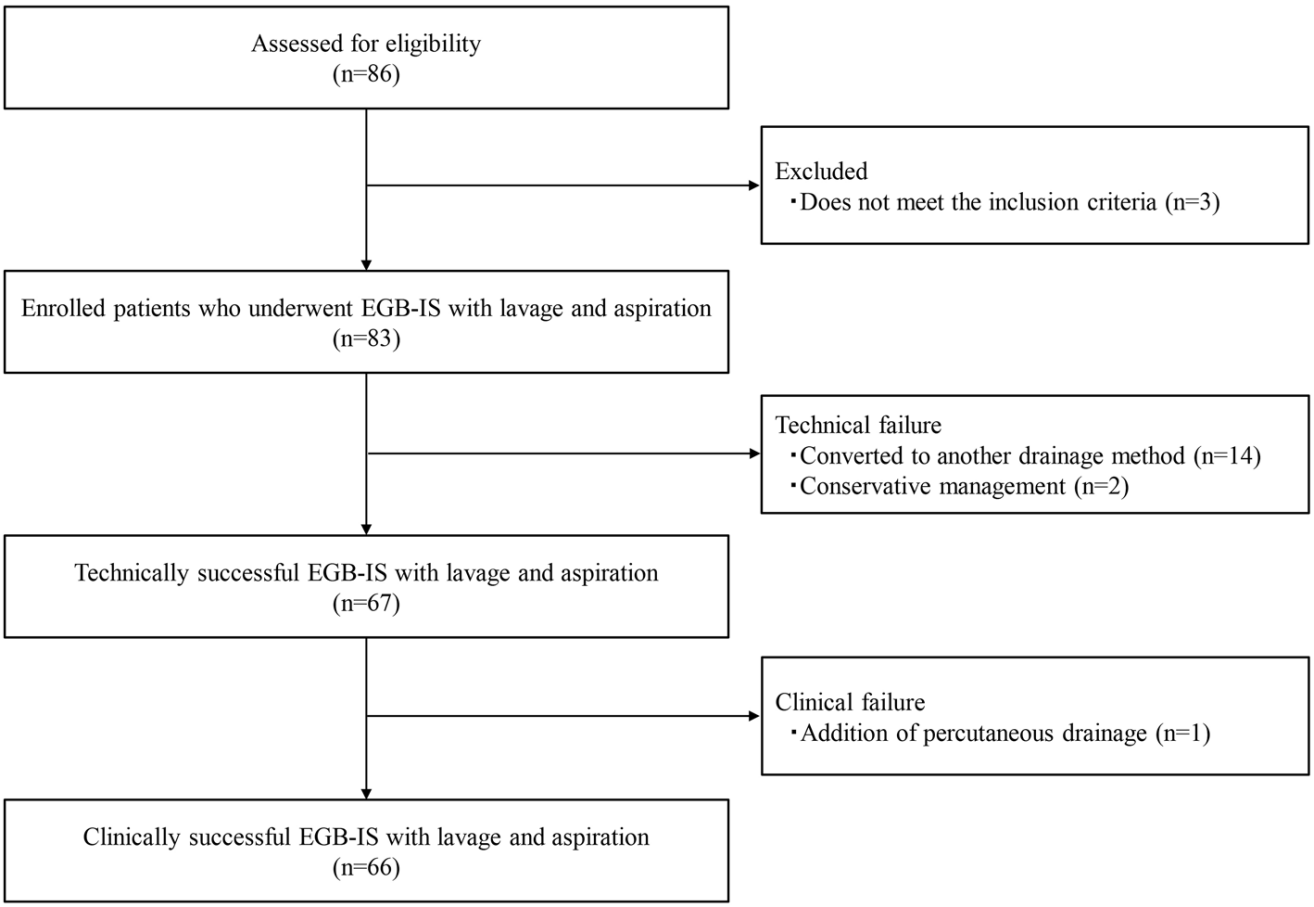
Flowchart detailing the enrollment and outcomes of the patients in this study. *EGB-IS* endoscopic gallbladder inside-stenting.

Table 2 depicts the outcomes of EGB-IS with lavage and aspiration. The technical success rate was 80.7% (67/83). The reason for technical failure of all cases was difficulty in reaching the gallbladder through the cystic duct. The median quantity of bile aspirated was 60 (IQR 50–100) mL, and the median quantity of saline-aspirated lavage was 100 (IQR 100–100) mL. The median procedural time was 35 (IQR 27–48) min. The clinical success rate in patients in whom technical success was achieved was 98.5% (66/67) (Fig. 4). The frequency of early AEs was 3.6% (3/83). Moderate pancreatitis occurred in two patients, and mild post-endoscopic sphincterotomy bleeding occurred in one patient.

**Table 2:** Outcomes of endoscopic gallbladder inside-stenting combined with lavage and aspiration.

Technical success, n (%)	67/83 (80.7)
Median quantity of bile aspirated, (IQR) (mL)	60 (50–100)
Median quantity of saline lavage, (IQR) (mL)	100 (100–100)
Median procedural time, (IQR) (min)	35 (27–48)
Clinical success, n (%)	66/67 (98.5)
Early-adverse events, n (%)	3/83 (3.6)
Post-ERCP pancreatitis	2
Post-EST bleeding	1
Recurrence of cholecystitis, n (%)	3/66 (4.5)
Late symptomatic AEs, except cholecystitis, n (%)	4/66 (6.1)
Cholangitis	3
Liver abscess	1
Overall late symptomatic AEs (cholecystitis + other events), n (%)	7/66 (10.6)

*IQR* interquartile range, *ERCP* endoscopic retrograde cholangiopancreatography, *EST* endoscopic sphincterotomy, *AE* adverse event.

### Long-term outcomes

The rate of incidence of recurrent cholecystitis was 4.5% (3/66), all instances of which were treated by removal of the stent using the thread and replacement with another stent. The rate of symptomatic late AEs, besides cholecystitis, was 6.1% (4/66). Cholangitis occurred in three patients, and liver abscess occurred in one patient. Cholangitis was treated by removal of the stent using the thread, followed by deployment of a bile duct stent. Liver abscess was treated with conservative management using antibiotics.

**Figure 5:**
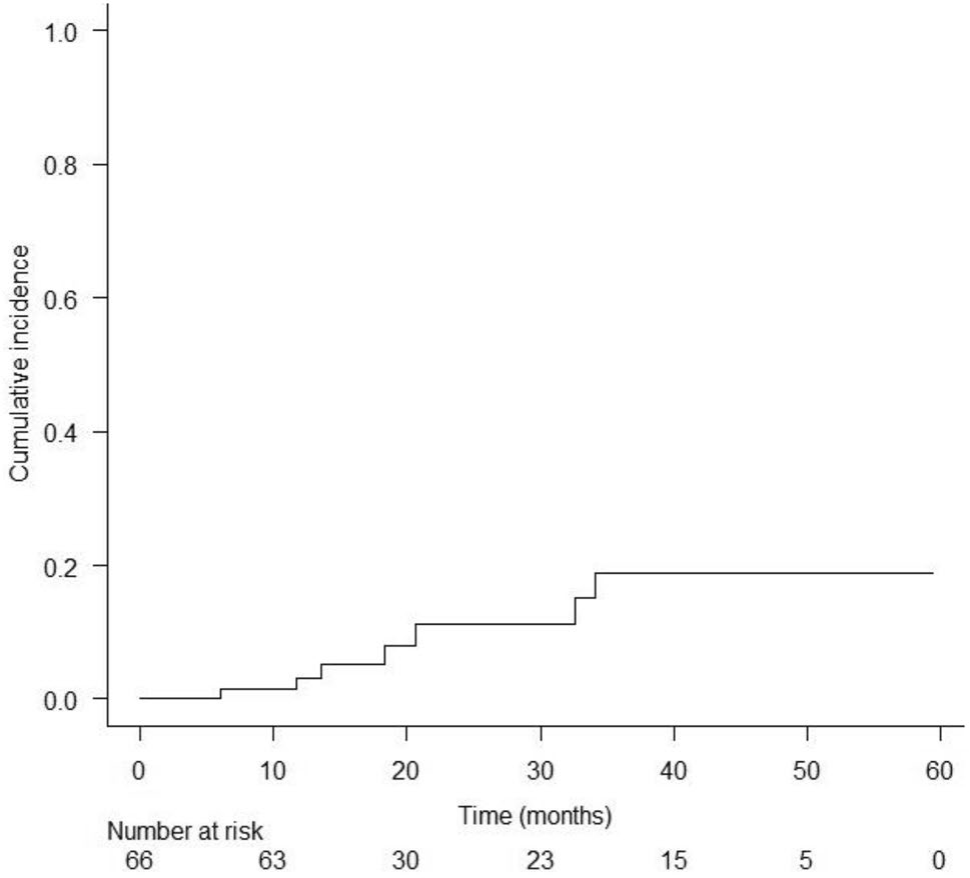
Cumulative incidence of overall symptomatic late adverse events (recurrent cholecystitis + other events) determined using Kaplan–Meier analysis. The 1-, 2-, and 3-year cumulative incidence rates of overall adverse events were 3.2%, 11.2%, and 18.9%, respectively.

Twenty-three patients died for reasons unrelated to the procedure. The rate of overall late AEs (cholecystitis plus other events) was 10.6% (7/66). Kaplan–Meier analysis revealed that the 1-, 2-, and 3-year cumulative incidence rates of overall late AEs were 3.2%, 11.2%, and 18.9%, respectively (Fig. 5).

## Discussion

This study demonstrated that the rate of recurrent cholecystitis and that of late AEs, other than cholecystitis, were low after EGB-IS with lavage and aspiration. Although the clinical success rate was good, the technical success rate was insufficient.

Prevention of recurrent cholecystitis in patients with calculous cholecystitis in whom cholecystectomy is contraindicated is an important issue in the treatment of cholecystitis^23^. The mechanisms by which long-term stent placement with EGBS prevents the recurrence of cholecystitis include the drainage effect within the stent, prevention of gallstone impaction by the stent, and straightening of the spiral structure of the cystic duct, which permits flow along the stent surface, even if the stent is occluded^24^. These mechanisms act in conjunction with each other to exert a preventive effect. Four comparative studies^11–14^ that investigated the long-term outcomes of EGBS reported that the rate of recurrent cholecystitis was 0–6.3%, which is significantly lower than that in patients who underwent percutaneous drainage (16.0–19.2%). On the other hand, the rate of late AEs, besides recurrent cholecystitis, i.e., mainly cholangitis, was reportedly high at 9.1–19.6%. A recent large-scale study^14^ indicated that the overall late AE rate (including cholecystitis plus other events) did not differ significantly between patients who underwent EGBS and those who underwent tube removal after PTGBD (25.9% vs. 30.0%, P = 0.500). Therefore, it is necessary to devise methods to reduce the frequency of other late AEs, mainly cholangitis, in order to optimize the preventive effect of EGBS against the recurrence of cholecystitis in poor surgical candidates. Retrograde infection due to duodenobiliary reflux, bacterial contamination, and cholestasis associated with the long-term presence of stents are thought to be the principal factors responsible for the onset of cholangitis^14,25^.

Sufficient lavage and aspiration can enhance the drainage effect and reduce the likelihood of stent occlusion, leading to better long-term outcomes, because infected bile is often viscous and turbid due to debris, which could also have contributed to the higher clinical success rate. Inside stenting can limit duodenobiliary reflux and adhesion of food residue to the distal end of the stent. Several studies on inside stenting for hilar biliary strictures have suggested that it results in significantly better long-term stent patency than that with stent placement across the papilla^26–28^. The present study is the first to investigate lavage and aspiration and the inside stent for gallbladder stenting: it found that the recurrence rate of cholecystitis was 4.5%, which is similar to previous reports of long-term stent placement via EGBS, and the rate of late AEs, other than cholecystitis, was 6.1%, which was lower than that reported in previous studies. Therefore, a certain effect may be expected when the inside stenting technique is employed for EGBS in conjunction with lavage and aspiration.

However, the low technical success rate is an important problem associated with EGBS. The bougie effect of the dedicated lavage catheter may also be effective in cases where passage of the stent through the cystic duct is difficult due to severe inflammation. However, the technical success rate in this study was 80.7%, which is approximately as insufficient as that reported in previous studies; thus, a sufficient additive effect of the catheter cannot be inferred. Guidewire selection for accessing the gallbladder through the cystic duct is a major factor responsible for failure of the procedure^29^. Therefore, even if the method described in this study improves long-term outcomes, an innovative maneuver that can improve the technical success rate is definitely required to ensure widespread application of EGBS as standard treatment in poor surgical candidates with calculous cholecystitis.

This study should be considered in the context of the limitations associated with its single-center setting, small sample size, and lack of a control cohort. Furthermore, the results may not be directly applicable to other institutions because all procedures were performed or supervised by a single endoscopist with experience in EGBS. Moreover, the AE rates could have been underestimated because the follow-up period may have been insufficient. The results of this study need to be confirmed by further clinical studies, including randomized controlled trials.

In conclusion, this is the first study to assess EGB-IS with lavage and aspiration for calculous cholecystitis in poor surgical candidates, with promising results, especially with respect to the long-term outcomes. This method may be a useful option when EGBS with long-term stent placement is planned, since the prevention of cholecystitis recurrence without an increase in the incidence of other AEs is anticipated.

## Data Availability

The datasets used and/or analyzed during the current study available from the corresponding author on reasonable request.

## Abbreviations

EGBS: Endoscopic gallbladder stenting
PTGBD: Percutaneous transhepatic gallbladder drainage
AE: Adverse event
EGB-IS: Endoscopic gallbladder inside-stenting
ASA-PS: American Society of Anesthesiology Physical Status
CCI: Charlson comorbidity index
IQR: Interquartile range
